# The rational design of affinity-attenuated OmCI for the purification of complement C5

**DOI:** 10.1074/jbc.RA118.004043

**Published:** 2018-07-20

**Authors:** Alex Macpherson, Xiaofeng Liu, Neesha Dedi, Jeffery Kennedy, Bruce Carrington, Oliver Durrant, Sam Heywood, Jean van den Elsen, Alastair D. G. Lawson

**Affiliations:** From the ‡ UCB-Celltech, Slough SL1 3WE, United Kingdom and; the § Department of Biology and Biochemistry, University of Bath, Bath BA2 7AX, United Kingdom

**Keywords:** complement system, mutagenesis, biophysics, protein purification, complement, complement component C5, immune evasion, OmCI

## Abstract

Complement component C5 is the target of the mAb eculizumab and is the focus of a sustained drug discovery effort to prevent complement-induced inflammation in a range of autoimmune diseases. The immune evasion protein OmCI binds to and potently inactivates C5; this tight-binding interaction can be exploited to affinity-purify C5 protein from serum, offering a vastly simplified protocol compared with existing methods. However, breaking the high-affinity interaction requires conditions that risk denaturing or activating C5. We performed structure-guided *in silico* mutagenesis to identify prospective OmCI residues that contribute significantly to the binding affinity. We tested our predictions *in vitro*, using site-directed mutagenesis, and characterized mutants using a range of biophysical techniques, as well as functional assays. Our biophysical analyses suggest that the C5–OmCI interaction is complex with potential for multiple binding modes. We present single mutations that lower the affinity of OmCI for C5 and combinations of mutations that significantly decrease or entirely abrogate formation of the complex. The affinity-attenuated forms of OmCI are suitable for affinity purification and allow elution under mild conditions that are nondenaturing or activating to C5. We present the rational design, biophysical characterization, and experimental validation of affinity-reduced forms of OmCI as tool reagents to enable the affinity purification of C5.

## Introduction

Complement component C5 is a large, 188-kDa protein that is integral to the complement system. As an eventual consequence of activation of any of the classical, lectin, or alternative pathways, C5 is cleaved into two functionally distinct moieties (1, 2). The smaller anaphylatoxin subunit C5a signals via C5aR, a pro-inflammatory G-protein–coupled receptor (3, 4). The cleavage of C5a triggers a major conformational change in the C5d–CUB–MG8 superdomain, forming the metastable C5b subunit (5). The conformationally primed C5b can then assemble with C6-C9 to form the membrane attack complex, which results in the targeted lysis of pathogenic or damaged cells (6–8).

Complement C5 has been targeted with various therapeutics, most notably by eculizumab, a mAb that binds to the MG7 domain of C5 (9, 10) preventing cleavage by both the alternative pathway C5 convertase (C3bBbC3b) and the classical and lectin pathways' convertase (C4bC2aC3b) (1, 2, 10–12). Eculizumab is approved for the treatment of paroxysmal nocturnal hemoglobinuria (13, 14) and atypical hemolytic uraemic syndrome (15, 16), and clinical studies are underway to expand into indications including age-related macular degeneration (17, 18), myasthenia gravis (19, 20), optic neuritis (21), and kidney transplant rejection (22, 23). Second generation therapeutics including affibodies, cyclic peptides, aptamers, and small molecules that target C5 are currently in clinical or preclinical development (24, 25).

Intriguingly, parasites have also developed strategies of targeting C5 to evade lysis by the complement system. Two species of tick, *Ornithodoros moubata* and *Rhipicephalus appendiculatus*, have independently evolved salivary proteins that inactivate C5 when secreted during feeding (9, 26–29). The two proteins, dubbed OmCI and RaCI after their respective species of origin, are of comparatively low molecular weight and are distinct in both sequence and structure.

A crystal structure of the C5–OmCI–RaCI ternary complex (9) shows that both proteins bind at opposing ends of the C5d domain of C5, anchoring it to neighboring domains. These sites are distant from the eculizumab and cobra venom factor–binding sites on the MG7 domain (10, 30) and may prevent C5 activation by a different inhibitory mechanism.

OmCI, a 17-kDa protein exhibiting the eight-stranded, antiparallel β-barrel fold characteristic of the lipocalin family, binds with the C5d and CUB domains of C5 (9, 26). Although it does not appear to have bonded interactions with the neighboring, highly mobile C345C domain, such is their proximity that OmCI may still influence its conformational sampling.

As a lipocalin, OmCI has evolved to interact with small hydrophobic molecules (31). In addition to preventing release of C5a, OmCI also sequesters the proinflammatory molecule leukotriene B4 (LTB4),^2^ making it a potent inhibitor of the early inflammatory response (27). A crystal structure of the OmCI–LTB4 complex shows the aliphatic chain of LTB4 bound within a conical binding cavity in the center of the β-barrel fold (28). The pocket is comparatively promiscuous, also binding palmitoleic and elaidic fatty acids. OmCI can bind LTB4 and C5 simultaneously in a noncooperative manner (28).

OmCI has broad cross-species reactivity and is being developed commercially in a modified form as Coversin (32), primarily targeting patients with the R885H mutation, a perturbation to the eculizumab epitope that renders them resistant to treatment with the mAb (33, 34).

Akin to C3 and C4, C5 is comparatively abundant in serum at a concentration of 75 μg/ml (35). It expresses poorly in recombinant systems and is usually purified from serum. The standard protocol involves a serum-precipitation step, followed by two sequential ion-exchange purifications and a gel-filtration step (36, 37). Alternative purification approaches have been described, including hydrophobic interaction chromatography and immunoadsorption (38, 39). Immunoadsorption is incumbent on the immunization of animals to generate a polysera that, unless a mAb is used, may not be a specific capture mechanism, with only a proportion of the sera being specific to C5 and with the potential for inconsistent performance caused by batch variation. An additional complication is that changes in pH have been shown to activate C5 and subsequently mild, nonactivating elution conditions must be found to break the interaction with the antibody (37).

The C5–OmCI interaction has been previously exploited to purify C5 and OmCI complexes for crystallography (9). OmCI expresses extremely well in bacterial, yeast, or mammalian cells, with a yield of 0.4 g/liter reported in yeast (28). It binds C5 with high affinity and, with suitable modifications to modulate affinity, could be an ideal recombinant tool with which to purify C5 from serum.

The crystal structure allows computational energy calculations to guide the selection of residues to be attenuated using mutations to alanine or glycine. These rationally designed mutants may display differing affinities but also changing susceptibility to pH or ionic conditions, which may be exploited to create a specific recombinant reagent for affinity purification. If discovered, these reagents would markedly simplify the purification of complement C5 and aid academic and industrial research.

## Results

### In silico design of affinity-impaired OmCI mutants

**Figure 1.**
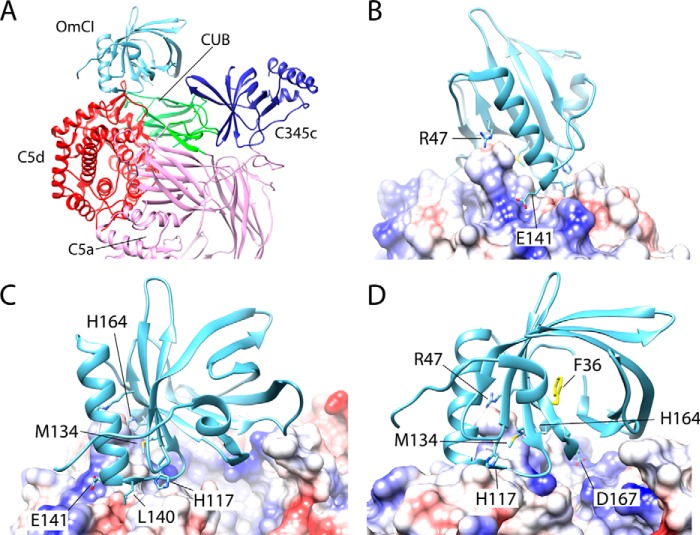
**Molecular interactions of the C5–OmCI complex.** *A* shows OmCI in complex with the C5d and CUB domains, on the C5d–CUB–MG8 superdomain of C5 (Protein Data Bank code 5HCC). *B* shows the surface of C5 colored by charge, with *red* denoting areas of negative charge and positively charged areas shown in *blue*. The two OmCI residues that contribute significantly to binding affinity, Glu-141 and Arg-47, are shown. *C* shows the residues identified from our *in silico* mutagenesis study as contributing significantly to binding affinity, with *D* showing the 180° view.

Using information from the crystal structure of the C5–OmCI–RaCI complex, we designed single residue mutations to reduce OmCI–C5 affinity using Molecular Operating Environment (MOE) software (40, 41) (Fig. 1). Mutations were ranked by the dAffinity score, a surrogate for binding free energy. Mutations predicted to lower the energy of the interaction more than 1 kcal/mol were found (Table 1).

**Table 1:** **Summary table of key data generated with OmCI proteins, showing the selection of a small panel of mutants for profiling as a purification ligand**

	*In silico* prediction	Single-cycle kinetics*^a^*	CD	DSC*^b^*		AP ELISA	Multicycle kinetics		
OmCI mutation	dAffinity score	Approximate fold change in *k*_off_	Loss of secondary structure (217 nm)	Δ*T*_m_	ΔΔ*H*	pIC_50_	*k*_on_	*k*_off_	*K_D_*
	*kcal/mol*			°*C*	% *refolded*	−*log m*	*m s*^−*1*^	*s*^−*1*^	*m*
Wildtype OmCI			No		71.7	≤9.0	5.88E + 05	<1.0E − 05	<1.0E − 10
F36W	0	2.6							
D167A	1.9	2.4							
H117A	2.3	1.1							
R47A	3.1	4.1	No	−4.8	69.1	≤9.0			
M134G	4.3	229.0	Yes	−8.2	23.9				
L140G	5.4	1.0							
E141A	5.8	29.1	No	−3.3	61.7	≤8.9	4.81E + 05	3.04E − 04	7.09E − 10
H164A	7.6	3.5	No	−1.6	57.7	≤8.8			
E141A/R47A	9.3	973.0	No	3.1	54.1	8.0	3.01E + 06	3.18E − 02	1.31E − 08
E141A/H164A	11.3	197.0	No	−1.8	47.8	8.5	1.13E + 06	4.06E − 03	4.09E − 09
E141A/L140G	12.8	31.3							
E141A/R47A/M134G	14.6	NB*^c^*							
E141A/R47A/L140G	15.4	1270.0							
E141A/R47A/H164A/L140G	20.4	5500.0							

*^a^* Single-cycle kinetics sensorgrams are shown in Fig. S1. A nominal fold change relative to WT *k*_off_ (approximately 1.0E + 05 ms^−1^) is shown to exemplify changes in stability of the OmCI–C5 complex.

**Figure 2.**
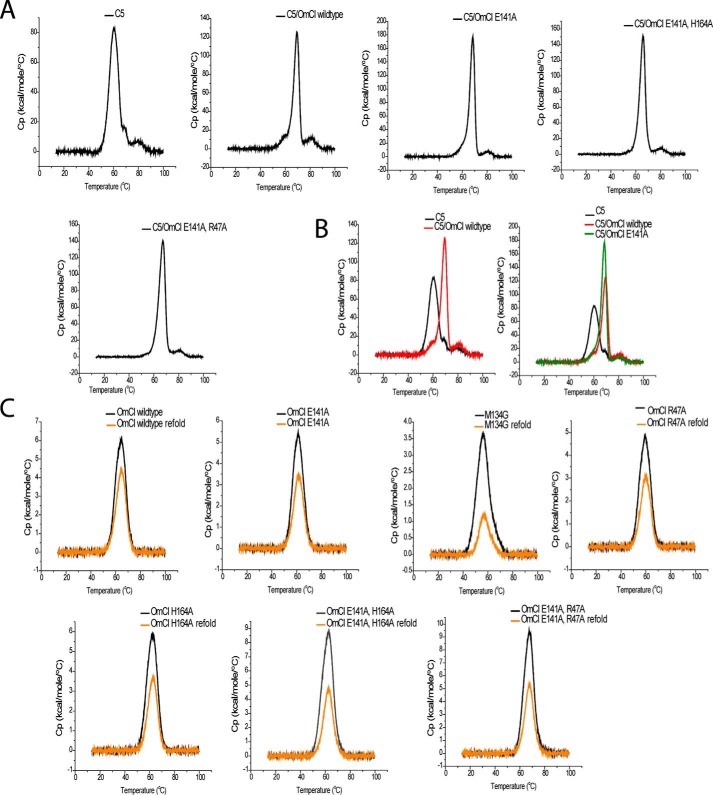
**DSC thermograms for C5 alone and in complex with OmCI proteins.** C5 displays a biphasic unfolding, the main peak has a slight shoulder and unfolds at 60.3 °C, with a smaller and more stable peak unfolding at 77.1 °C. Complexing C5 with OmCI stabilizes the main peak, in proportion to the binding affinity (*A*). A smaller stabilization effect, Δ*T*_m_ = +3 °C, is observed on the second peak but without clear correlation to the affinity of OmCI construct (*B*). *C* shows DSC thermograms for OmCI proteins. The first round of melting is shown in *black* with the second melt shown in *orange*. The peak area corresponds to the enthalpy of unfolding (Δ*H*) and by taking a ratio of the two peaks a ΔΔ*H* or percentage of refolding can be calculated.

*^b^* Δ*T*_m_ values are relative to the WT OmCI protein. The data are shown in Table S1 and Fig. 2.

*^c^* NB, no binding detected.

Notably, our analysis has selected charged residues that appear to participate in salt bridges or hydrogen bonds. Two hydrophobic residues, Met-134 and Leu-140, were also identified. When mutating hydrophobic residues, we chose glycine mutations rather than alanine to make a nonclashing mutation where hydrophobicity was entirely absent from the side chain.

### Validation of affinity-impaired OmCI mutants

To test our predictions, we made the following single residue mutations: R47A, H117A, M134G, L140G, E141A, H164A, and D167A. In addition, a control F36W mutation was made. This mutation is patented as abrogating LTB4 binding without altering the affinity for C5 (42). We tested these OmCI mutants by SPR in single-cycle kinetics experiments, with the aim of identifying a smaller panel of mutants to profile as a purification reagent. Single-cycle kinetics is performed by sequentially injecting increasing concentrations of analyte over the sensor chip, in the absence of any regeneration steps to remove bound analyte from the previous injection.

The sensorgrams could be fitted using a single-site binding model but were better explained by including additional parameters in a heterogenous ligand model. When using the single-site binding model, comparatively low standard errors (S.E.), <0.2%, were observed when fitting the rate constants. However, visually, the model appeared to poorly estimate the on rate (*k*_on_), overestimating the *k*_on_ at lower concentrations and underestimating at higher concentrations (Fig. S1).

Accordingly, we opted to rank the mutations based on changes in off rate (*k*_off_), which was paramount for affinity purification (Table 1). Two mutations, E141A and M134G, had a significant effect on the stability of the OmCI–C5 complex. The E141A mutation increased the *k*_off_ value of the complex by ∼29-fold and the M134G mutation by ∼229-fold. However, we were concerned that the M134G mutant displayed low binding, relative to the amount of immobilized material, suggesting that the protein had either been expressed with low activity or partially inactivated through amine coupling to the sensor chip. Other mutations had only modest effects on *k*_off_. The control F36W mutation, which is distal to the interface with C5, showed a small increase in *k*_off_ value and displayed the same stoichiometric ratio of binding as WT OmCI. Our sensorgrams suggest that the OmCI–C5 interaction is complex, and we chose to investigate further in multicycle kinetics experiments, which are described later.

### Designing a suitable C5 purification reagent ligand based on combined OmCI mutations

To further lower affinity, we modeled combinations of mutations. Again, we used MOE to simulate changes in binding energy (Table 1). Because of the potential for synergy, we retained mutations that showed little effect in this second round of designs and based our final selection on the output of our MOE analysis.

As predicted, combining mutations further abridge and, in some cases, entirely abrogate binding to C5. Combining either the H164A or the R47A mutation with E141A produced OmCI variants with *k*_off_ 197–973-fold faster than WT OmCI, characteristics favorable for application as affinity-purification reagents, retaining fast association with C5, but with markedly increased dissociation.

The largest effect was observed in the E141A/R47A/H164A/L140G quadruple mutant, which produced a square wave sensorgram, more characteristic of the fast binding kinetics of a low-molecular weight chemical fragment than a protein–protein interaction. The *k*_off_ value of this protein was ∼5,500-fold higher than WT OmCI. The M134G mutation completely prevented binding when used in combination with E141A and R47A.

### Structural and functional analysis

#### 

##### Circular dichroism

To look for gross changes in the secondary structure, we used CD. We tested the E141A/R47A and E141A/H164A double mutants, alongside their constituent single mutants: E141A, R47A, and H164A (Table 1). The M134G mutant, which markedly increased *k*_off_ value but significantly lowered binding, was also tested. The CD traces were closely comparable with WT OmCI, except for M134G, which showed a marked loss of structure at 217 nm consistent with an increase in random coil. Loss of the positive peak at 190 nm was also observed, suggesting a loss of overall secondary structure (Fig. S2).

##### Differential Scanning Calorimetry

We assessed the thermal stability of the mutants, their ability to refold after denaturation, and their capacity to stabilize C5 when complexed using differential scanning calorimetry (DSC). C5 displays a biphasic unfolding; the main peak unfolded at 60 °C, displaying a shoulder at 69 °C, whereas a smaller, more stable domain or conformer unfolded at 77 °C (Table 2 and Fig. 2). Upon complexing with OmCI, the main peak was stabilized significantly with a Δ*T*_m_ +8.6 °C. The mutants also increase the thermal stability of the main peak, with the degree of stabilization reducing in agreement with their attenuated affinity.

**Table 2:** **DSC data on C5–OmCI complexes** The data are from *n* = 1 experiment.

Sample	Peak 1 *T*_m_	Peak 1 Δ*H*	Peak 2 *T*_m_	Peak 2 Δ*H*
	°*C*	*cal/m*	°*C*	*cal/m*
C5	60.31 ± 0.012	8.1E + 05	77.06 ± 0.21	1.1E + 05
C5–WT OmCI	68.90 ± 0.020	7.1E + 05	81.36 ± 0.21	7.8E + 04
C5–E141A OmCI	67.99 ± 0.019	9.6E + 05	80.83 ± 0.33	8.1E + 04
C5–E141A/R47A OmCI	66.68 ± 0.016	9.1E + 05	80.96 ± 0.28	5.6E + 04
C5–E141A/H164A OmCI	65.41 ± 0.011	8.8E + 05	80.49 ± 0.27	5.3E + 04

The second peak may be stabilized upon OmCI binding, but the effect is smaller. The area under the peak, corresponding to the enthalpy of unfolding remains broadly the same, and the relationship to the affinity of the OmCI construct used is not as clear as observed with stabilization of the main peak.

The OmCI mutants are themselves typically less thermally stable than WT OmCI (Table 1, Fig. 2, and Table S1). The most destabilizing OmCI mutation was M134G, followed by R47A and E141A. The remaining mutants had comparatively modest Δ*T*_m_ of less than −3 °C. Of note, the E141A/R47A double mutant was more thermally stable than WT OmCI, despite the single R47A and E141A mutations being among the most destabilizing.

We performed a cycle of cooling prior to a second melt to assess the ability of the proteins to refold after denaturation. The peak area corresponds to the enthalpy of unfolding (Δ*H*), and by taking a ratio of the two peaks, a ΔΔ*H* or percentage of refolding can be calculated. As a large, complex protein with numerous disulfide bonds, C5 was unable to refold, but a proportion of OmCI could recover and be remelted at a *T*_m_ almost identical to the first cycle. There was an approximately 72% recovery for WT OmCI with 47–69% recovery of the mutants. Consistent with the CD data, the M134G mutant was largely unable to refold with only 24% of protein recovered.

##### SPR: Multicycle kinetics

Because of our observations when fitting data in our single-cycle kinetics experiments, we looked for further evidence of complex binding kinetics in the C5–OmCI interaction. To obtain more detailed kinetic data for our analysis, we performed multicycle kinetics experiments by SPR. Multicycle kinetics performs a single injection of a single sample concentration within a cycle, regenerating the surface after each injection to dissociate bound material. To exclude heterogeneity in the immobilization steps, OmCI was uniformly immobilized in a fixed orientation, using biotin conjugated to an N-terminal AVI tag.

**Figure 3.**
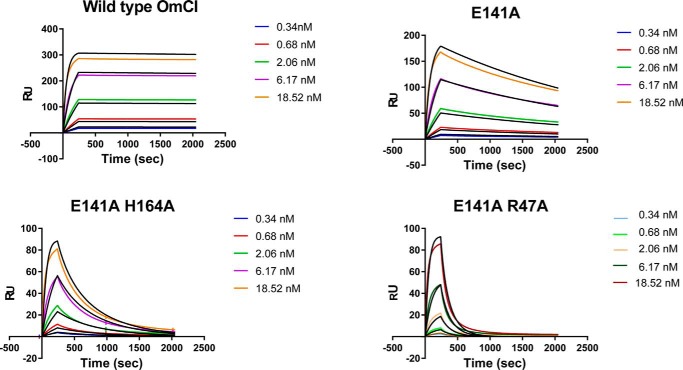
**Sensorgrams from multicycle kinetics.** The sensorgrams above show raw data (colored by concentration) and the fit from a single-site binding model (in *black*). The mutants display an accelerated dissociation relative to WT OmCI. Fitting with the single-site model appears to underestimate *k*_on_ at low concentrations, whereas at higher concentrations it appears to overestimate it.

These data were explained by a single-site binding model, with the fitting of *k*_on_ generating broadly acceptable S.E. values. The refractive index changes upon binding did not suggest that the binding stoichiometry exceeded 1:1. However, consistent with our single-cycle experiments, the single-site binding model again appeared to underestimate *k*_on_ at low concentrations and overestimate at higher concentrations (Fig. 3). This suggests that observations in single-cycle kinetics experiments were not the result of heterogenous immobilization to the sensor chip and may indicate additional complexity in the OmCI–C5 binding interaction.

We report individual rate constants and *K_D_* values for the mutant OmCI proteins binding to C5 (Table 1, Fig. 3, and Table S2). The WT protein exhibited antibody-like affinity, exceeding the 1.0E−5 s^−1^ limit for accurate determination of *k*_off_ by SPR (43). In our experiments, the *K_D_* of WT OmCI is <100 pm, at least 10-fold lower than previously reported (28). The E141A single mutant displayed a >7-fold decrease in affinity (*K_D_* of 0.7 nm), and the E141A/H164A double mutant displayed a >40-fold decrease in affinity (*K_D_* of 4 nm). Finally, the E141A/R47A double mutant displayed a >130-fold decrease in affinity (*K_D_* of 13 nm), relative to WT. Importantly, these decreases in affinity were mediated by increases in the *k*_off_ values.

##### Complement activation ELISA

We tested the mutants in an alternative pathway (AP) activation ELISA to establish the extent to which affinity-abridged forms of OmCI could inhibit formation of the MAC (Table 1, Table S3, and Fig. S3). The single mutants and WT OmCI were potent inhibitors, displaying steep Hill slopes, indicating that they had reached the tight binding limit of the assay. The double mutants E141/R47A and E141A/H164A showed >10- and >5-fold losses of potency, respectively. Despite the variation in potency, all the mutants could effectively inhibit formation of the MAC at sufficiently high concentrations.

### Affinity purification of C5 with the E141A/H164A double mutant

Having engineered faster dissociating forms of OmCI, we began to develop methods for the affinity purification of C5 from serum. OmCI proteins were biotinylated via an N-terminal tag and captured on a streptavidin column. Fractionated serum was applied to the column and conditions for the elution of C5 were tested.

With WT OmCI (*k*_off_ = <1.0E−5 s^−1^), C5 was captured but would not elute with either 2 m NaCl or 3 m MgCl_2_. Using a pH gradient, C5 was eluted at approximately pH 2.8, where it formed a milky precipitate, which could be reversed upon neutralization with 1 m Tris.

Working to the “Goldilocks principle,” we hoped to identify a mutant with the ideal kinetic profile to permit capture of C5 but with a suitably attenuated *k*_off_ to allow the interaction to be destabilized and eluted under mild conditions. We ran small scale purifications, trialing the mutants as capture reagents in order of their faster dissociation. The E141A single mutant (*k*_off_ = 3.0E−04 s^−1^) would not permit elution with either NaCl or MgCl_2_. The abridged affinity had only a modest effect on the pH gradient, with C5 still eluting at a pH of ∼2.8.

The E141A/H164A double mutant (*k*_off_ = 4.0E−03 s^−1^) was the next mutant trialed as an affinity-purification reagent. With this mutant, C5 was captured and then eluted with either 2 m NaCl or 3 m MgCl_2_. C5 did not respond favorably to elution with high NaCl, irreversibly precipitating when eluted at 2 m and eluting at 1 m in a broad, flat peak. Conversely, 3 m MgCl_2_ gave a sharp, symmetrical elution peak with no visible precipitation in the fractions. We tested lower concentrations of MgCl_2_ and found we could elute C5 using a 2 m solution, but with a 1 m solution tailing of the peak was observed.

**Figure 4.**
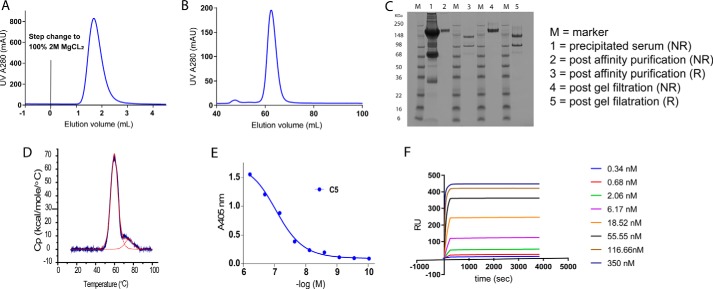
**Purification of functional C5 with affinity-attenuated E141A/H164A OmCI mutant.** *A* shows the elution of C5 from an E141A/H164A OmCI column using isocratic elution with 2 m MgCl_2_, after one column volume the material elutes in a sharp and broadly symmetrical peak. *B* shows elution of affinity-purified C5 from a gel-filtration column as a single peak with a small high-molecular weight peak. *C* is a reduced and nonreduced SDS–PAGE gel of the material, before and after affinity purification and gel filtration (5 μg of the final C5 sample is loaded). A thermal melt of the protein by DSC is shown in *D*, with a *T*_m_ of 59.9 °C for the main peak. The ability of the material to restore complement activation to C5-depleted serum, with a pEC_50_ of −7.1 −log M, is shown in *E*. Finally, binding of our purified C5 to WT OmCI is shown in a multicycle kinetics experiment, with a *K_D_* <100 pm in *F*.

After a gel-filtration step, we tested the purified C5 to ascertain its quality and functional activity (Fig. 4). The final material was pure by SDS–PAGE, the thermal melt was consistent with commercially available C5 by DSC, and it bound to WT OmCI with high affinity, <100 pm, in a multicycle kinetics experiment. Crucially, the material was functionally active and restored activity to C5-depleted serum in an AP activation ELISA, with a pEC_50_ of 7.1 −log M. The E141A/R47A double mutant (*k*_off_ = 3.2E−02 s^−1^) proved to have too fast a dissociation rate to retain C5 on the column prior to elution.

## Discussion

*In silico* and biophysical analysis of the C5–OmCI interaction has identified residues that contribute significantly to the high binding affinity. We show that the OmCI–C5 interaction is complex and higher affinity than previously thought, and we demonstrate the utility of affinity-attenuated OmCI as a purification reagent. The *in silico* mutagenesis, performed using MOE, identified seven residues that could significantly alter binding energy, including five charged residues that potentially contribute to the formation of either salt bridges or hydrogen bonds.

The C5–OmCI–RaCI crystal structure suggests there are two ionic interactions between C5 and OmCI, arising from a bidentate salt bridge between Glu-141_OmCI_ and Arg-1226_C5_. Experimentally, E141A was the most effective single mutation, which highlights the importance of the salt-bridge interactions.

The other polar residues lack suitably charged opposing residues on C5 with which to make ionic interactions and instead make interchain hydrogen bonds. In the structure, His-164_OmCI_ and His-117_OmCI_ appear to make hydrogen bonds with the backbone carbonyl groups of Pro-1221_C5_ and Ser-1236_C5_, respectively. Two residues appear to make bidentate hydrogen bonds: Asp-167_OmCI_ with the backbone amide groups of Arg-955_C5_ and Arg-956_C5_; and Arg-47_OmCI_ with the side chain carbonyl and amide groups of Asn-1221_C5._

Experimentally, the rank order of affinity of the hydrogen bonding residues was broadly as per the predictions in MOE, with the *K_D_* of H164A < D167A < H117A. However, our analysis in MOE overestimated the impact of the H164A mutation relative to removal of the E141 salt bridge and underestimated the importance of the R47A mutation. The H164A mutation was predicted to be the most attenuating single mutation, but it was found to be less effective than either E141A or R47A.

Two hydrophobic residues, Met-134_OmCI_ and Leu-140_OmCI_, were also identified in the MOE analysis. Both are adjacent to charged residues (Asp-135_OmCI_ and Glu-141_OmCI_, respectively), and may have been identified as either lowering the desolvation or conformational entropy of their polar neighbors. We had predicted that L140G would be the third most effective mutation, but as a single mutation, it was largely ineffective and made only a modest contribution when used in combination. The single M134G mutation virtually attenuated binding, but CD and DSC data, suggest this is likely to be due to instability arising from a loss of secondary structure. Met-134 is at the end of a β-sheet, and the insertion of a flexible glycine residue may be poorly tolerated in this position.

Met-134 marks the end of BH-α3, a predominantly hydrophobic loop on which L140G is situated, connecting the large α3 helix of OmCI to the β-barrel fold. In complex with C5, this loop nestles in a hydrophobic channel on the C5d domain, bordered by Ala-1216_C5_–Val-01218_C5_ and Ser-123_C5_–Val-1239_C5_. Although the region is hydrophobic, interchain hydrogen bonds appear to form between the backbone moieties. These are not side-chain interactions and subsequently could not be easily investigated by mutagenesis, but they may still contribute significantly to the free energy of binding, with the hydrophobic environment ensuring a low desolvation entropy.

Finally, the control mutation, F36W_OmCI_, showed only a slight increase in *k*_off_ value, as expected. Although this mutant still displayed high affinity binding to C5, this may indicate a subtle allosteric effect arising from this distal mutation.

Overall the structure-based *in silico* approach was effective in designing mutations, reducing the number of mutants that had to be made and tested. Although there were some notable outliers in the ranking, there is a reasonable correlation with our experimental data.

When considering binding modes, our single-cycle kinetics experiments suggested the potential for a complex interaction that although displaying refractive index changes consistent with a 1:1 stoichiometry was not best described with a one-site binding model. We performed multicycle kinetics, with site-specific immobilization of OmCI via an N-terminal tag and observed similar outcomes when fitting data using the single-site binding model, suggesting that the complex kinetics are not the result of a heterogenous immobilization. Both the C5d and CUB domains interact with OmCI, and this creates the potential for multiple binding modes should an interaction with either domain be made first.

C5 displays a biphasic unfolding when thermally denatured. A main peak unfolds at 60 °C, with a shoulder at 69 °C, and a second smaller unfolding occurs at 77 °C. The shoulder of the main peak observed in C5 may be of interest, because it closely matches the Tm of the main peak in the C5–WT OmCI complex. This may suggest that OmCI is selecting a naturally occurring C5 conformation in which C5 is resistant to cleavage by the C5 convertases.

The smaller unfolding at 77 °C may also arise from a conformer of C5. If this is the case, the small size of the peak, corresponding to the enthalpy of unfolding, would suggest that the conformation is infrequently sampled. Although OmCI may bind to and stabilize the conformer, increasing *T*_m_, the Δ*H* remains broadly consistent, which suggests it is not a conformation that OmCI selects for or induces.

Alternatively, this could be a more stable domain that is structurally distinct from the MG domains, which constitute a large proportion of C5, potentially either the C5d, CUB, or C345C domains. Complexing with OmCI appears to stabilize this domain by +3 °C, but there is not a clear correlation to affinity when complexed with mutant OmCI. The lack of correlation to affinity could indicate an interaction between C5–OmCI that is not within the binding interface, as seen in the crystal structure, and therefore is not attenuated by our mutations provided full occupancy is maintained.

When looking at the charge profile of the surface of C5 and OmCI, we noted a positively charged cavity between the C5d and CUB domains (Fig. S4). The surface of OmCI is negatively charged, and there may be further complementary charge interactions with this cavity that are prevented by Phe-1631_C5_ protruding from the C345C domain and occluding the cavity (Fig. 1*A*). The position and proximity of the flexible C345C domain appears to be the result of crystal contacts between C5 molecules, rather than a specific interaction with the OmCI protein, suggesting that this positively charged region on C5 may be accessible in solution.

When testing our mutants in AP ELISA, we did not see reductions in *E*_max_ as a result of removing contacts, which would have indicated allosteric inhibition. Our mutants behave in the functionally competitive manner of the WT, which could indicate a competitive or steric mechanism for OmCI inhibition.

Our affinity-attenuated forms of OmCI are useful tools for the purification of C5; they are highly expressed and able to refold after complete denaturation at 100 °C. It is fortunate that the mutations decreased *k*_off_ specifically, with only modest effects on *k*_on_, and as such are ideal capture reagents for affinity columns. Our final purification reagent, E141A/H164A OmCI, is thermally stable and resilient to denaturation. The elution conditions this mutant has enabled are comparatively gentle, with no evidence of C5 activation with short-term exposure to 2 m MgCl_2_. This reagent will be a valuable tool for the purification of C5 to support research and drug discovery.

## Experimental procedures

### Design of mutations

MOE (2016.08) was used to virtually mutate individual OmCI residues to alanine in the crystal co-complex structure of OmCI–C5 (Protein Data Bank code 5HCC). The effect of these changes on the C5 interaction were scored using a binding free-energy prediction (dAffinity). Alanine and glycine mutations that lowered dAffinity by more than 1 kcal/mol were selected for experimental validation.

### Construct design

A DNA sequence, comprising the WT OmCI reference sequence (Uniprot entry Q5YD59), with a N-terminal AVI-tag peptide sequence (GLNDIFEAQKIEWHE) and polyhistidine tag, was designed using Vector NTI. In total, 15 gene variants were designed with single or double mutations to validate the *in silico* affinity predictions. The amino-acid sequence of the OmCI proteins can be found in Fig. S5. Custom synthesis and cloning into a mammalian expression vector was performed by ATUM.

### Small-scale expression and purification

Plasmid DNA for each construct was amplified using Qiagen Plasmid Plus Giga kits and quantified by *A*_260_. Individual 50-ml ExpiHEK 293 cell cultures, at 3 × 10^6^ cells/ml, per construct, were set up using Expifectamine 293 Transfection kits (Invitrogen), as per the manufacturer's instructions. The cells were cultured for 4 days and centrifuged at 4000 rpm for 1 h.

The supernatants were filtered through a 0.22-μm sterifilter, and each sample was purified on 1.0 ml of nickel-Sepharose Excel capture resin (GE Healthcare). The following modifications were made to the manufacturer's protocol: after binding, affinity captured protein was washed with 20× resin volume using buffer A (0.5 m NaCl, 0.02 m imidazole, PBS, pH 7.3). Protein samples were then eluted using buffer B (0.5 m NaCl, 0.25 m imidazole, PBS, pH 7.3) as ten 2-ml fractions. Postelution, the proteins were concentrated, and buffer was exchanged using 10-kDa spin concentrators (MerckMillipore). The protein concentration was determined by *A*_280_, and the samples were analyzed by SDS–PAGE before storage at −80 °C.

### Bir-A biotinylation of AVI-tagged OmCI

Biotinylation of the n-terminal AVI tag was performed using a Bir-A labeling kit (Avidity), as per the manufacturer's instructions. OmCI protein was buffer exchanged in 10 mm Tris, pH 8.0, using 2-ml Zeba desalting columns (Thermo Fisher) and diluted to 40 μm. Bir-A enzyme was added at 11.625 μg/ml, and BioMix B was added at 10% (v/v). An overnight incubation at room temperature was performed. Prior to use, unreacted biotin was removed using a PD-10 desalting column (GE Healthcare).

### Surface plasmon resonance: Single-cycle kinetics

Experiments were performed using a Biacore 8K (GE Healthcare). The OmCI mutants were immobilized on a CM5 chip using amine coupling. To achieve a minimal immobilization both flow cells were activated using EDC/NHS at a 1:2 molar ratio (flow rate, 10 μl/min; contact time, 30 s). Solutions of OmCI at 1 μg/ml were prepared in pH 4.5 Sodium-acetate buffer and immobilized on flow cell two only (flow rate, 10 μl/min; contact time, 420 s). Finally, ethanolamine was applied to both flow cells (flow rate, 10 μl/min; contact time, 420 s). A final immobilization level of ∼50 response units was obtained.

Single-cycle kinetics were measured using a 9-point, 3-fold titration of C5 (CompTech) from 500 nm in HBS-EP buffer. A high flow rate of 40 μl/min was used, with a contact time of 230 s and a dissociation time of 900 s. Binding to the reference surface was subtracted, and the data were fitted to a single-site binding model using Biacore evaluation software.

### Differential scanning calorimetry

DSC was performed using a Malvern Microcal VP Capillary DSC. Samples of C5 (CompTech) and OmCI protein were diluted to 1 mg/ml in PBS. For complexing experiments, C5 and OmCI were mixed in 1:1.25 molar ratio and incubated for a minimum of 6 h at 4 °C. Samples were heated from 10 °C to 100 °C at a rate of 1 °C/min. The injection speed was 50 μl/s with three filling strokes used. Six buffer reads were measured prior to running samples, and a clean, consisting of three cycles of 20-min detergent incubations at 80 °C, was performed after each sample. For refolding experiments, after cooling for 1 h, a single rescan was performed under the same analysis parameters. The ;Data were analyzed using Origin 7.0 software and fitted using a non–two-state, cursor-initiated model.

### Circular dichroism

OmCI protein was buffer exchanged into 20 mm phosphate, pH 7.4, 150 mm NaF using 0.5-ml Zeba desalting columns (Thermo Fisher). CD spectra were acquired using a scan range of 185–260 nm, with a step size of 0.5 nm, a time-per-point of 1 s, and a bandwidth of 1 nm. The concentration of protein was 0.3 mg/ml, and the cuvette pathlength was 1.0 mm. An air blank was measured and automatically subtracted, and a buffer blank was measured and manually subtracted from the spectra.

### Surface plasmon resonance: Multicycle kinetics

Multicycle kinetics were measured using a Biacore 8K (GE Healthcare). Site-specifically labeled OmCI protein was diluted to 5 nm in HBS-EP. Both flow cells of an SA streptavidin chip were prepared by performing three sequential injections of 1 m NaCl, 20 mm NaOH (flow rate, 10 μl/min; contact time, 60 s). OmCI was then injected over flow cell 1 (flow rate, 10 μl/min; contact time, 60 s). Final immobilization levels in the range of 20–100 response units were obtained. A wash of the fluidics with a 1:1 mixture of isopropanol and 1 m NaCl, 20 mm NaOH was performed, prior to running samples.

A 7-point, 3-fold titration of C5 (CompTech) was prepared in HBS-EP buffer from 250 nm. This was injected at a flow rate of 30 μl/min, with a contact time of 240 s and a dissociation time of 3600 s. After each injection, the surface was regenerated with two sequential injections of 0.1 m citric acid monohydrate (pH 2.0) with a contact time of 30 s and flow rate of 30 μl/min.

Binding to the reference surface was subtracted, and the data were fitted to a single-site binding model, using Biacore evaluation software. When fitting data, curves at concentrations >18.5 nm (20–200×, *K_D_*) were excluded from the analysis to maximize curvature and increase the accuracy of fitting *k*_on_.

### Complement activation ELISA

An alternative pathway complement ELISA (Wieslab) was run as per the manufacturer's instructions. 10-point, 3-fold titrations of OmCI were made from 5 μm in human serum (TCS Bioscience) and allowed to incubate for 30 min at room temperature, prior to receiving a 118 dilution into assay diluent. The diluted samples were immediately plated onto the assay plate.

For experiments with purified C5, C5 was titrated from 5 μm in C5-depleted serum (Comptech), immediately diluted 118 into assay diluent, and plated. The data were analyzed using a four-parameter logistic fit in Prism 7.02.

### Ammonium-sulfate precipitation

Human serum (TCS Bioscience) was thawed in a 37 °C water bath. The serum was cooled to 4 °C, and 60 ml of a saturated ammonium sulfate solution was added dropwise per 100 ml of serum, with gentle stirring. The precipitate was collected by centrifugation at 10,000 × *g* for 30 min at 4 °C. The pellet was resuspended to the original serum volume using PBS, 10 mm EDTA, and a second round of precipitation was performed. Prior to loading on the column, the sample was filtered through a 0.22-μm sterifilter.

### Purification of C5 using affinity-attenuated OmCI

Using an Akta pure (GE Healthcare), biotinylated E141A/H164A OmCI was diluted to 20 μm, and 2.0 ml was injected onto a 1-ml Hi-Trap streptavidin HP column (GE Healthcare) at a flow rate of 0.5 ml/min.

The column was equilibrated with 5× column volume of PBS. Ammonium sulfate precipitated serum was loaded at a flow rate of 0.5 ml/min, and a 5× column volume wash with PBS at a flow rate of 1 ml/min was performed. C5 was eluted and collected in fractions, using an isocratic elution of 5× column volume of 20 mm Tris, 2 m MgCl_2_, at a flow rate of 1 ml/min.

Immediately after elution, the fractions were pooled and injected onto a HiLoad 16/600 Superdex 200-pg gel-filtration column (GE Healthcare) that had been pre-equilibrated with PBS. The column was run at 1 ml/min, and all peaks were collected. The peaks were analyzed by SDS–PAGE, and those with a molecular weight consistent with C5 monomer were pooled and stored at −80 °C.

## Author contributions

A. M., X. L., and A. D. G. L. conceptualization; A. M. data curation; A. M., X. L., and J. v. d. E. formal analysis; A. M. investigation; A. M. visualization; A. M., N. D., J. K., B. C., O. D., S. H., and A. D. G. L. methodology; A. M., J. v. d. E., and A. D. G. L. writing-original draft; A. M. project administration; A. M., X. L., N. D., J. K., B. C., O. D., S. H., J. v. d. E., and A. D. G. L. writing-review and editing; J. v. d. E. and A. D. G. L. supervision.

## Supplementary Material

Supporting Information

## Footnotes

2 The abbreviations used are:

LTB4: leukotriene B4
DSC: differential scanning calorimetry
MOE: Molecular Operating Environment
AP: alternative pathway.
