# Supplementary material for: The rational design of affinity-attenuated OmCI for the purification of complement C5
Source: J Biol Chem. 2018 Jul 20;293(36):14112–21. doi: 10.1074/jbc.RA118.004043 (PMC6130949; doi:10.1074/jbc.RA118.004043)
Supplement: Supporting Information [file supp_293_36_14112__index.html]

The rational design of affinity attenuated OmCI for the purification of Complement C5 — The rational design of affinity attenuated OmCI — The rational design of affinity-attenuated OmCI for the purification of complement C5 — The rational design of affinity-attenuated OmCI — Supporting Information 

# The rational design of affinity-attenuated OmCI for the purification of complement C5

## Supporting Information

- The rational design of affinity attenuated OmCI for the purification of Complement C5 - Supplementary Information - supplementary figures and data tables
