# Supplementary material for: The rational design of affinity-attenuated OmCI for the purification of complement C5
Source: J Biol Chem. 2018 Jul 20;293(36):14112–21. doi: 10.1074/jbc.RA118.004043 (PMC6130949; doi:10.1074/jbc.RA118.004043)
Supplement: Supporting Information [file supp_RA118.004043_138260_1_supp_162487_pb8z2n.pdf]

Supplementary Information

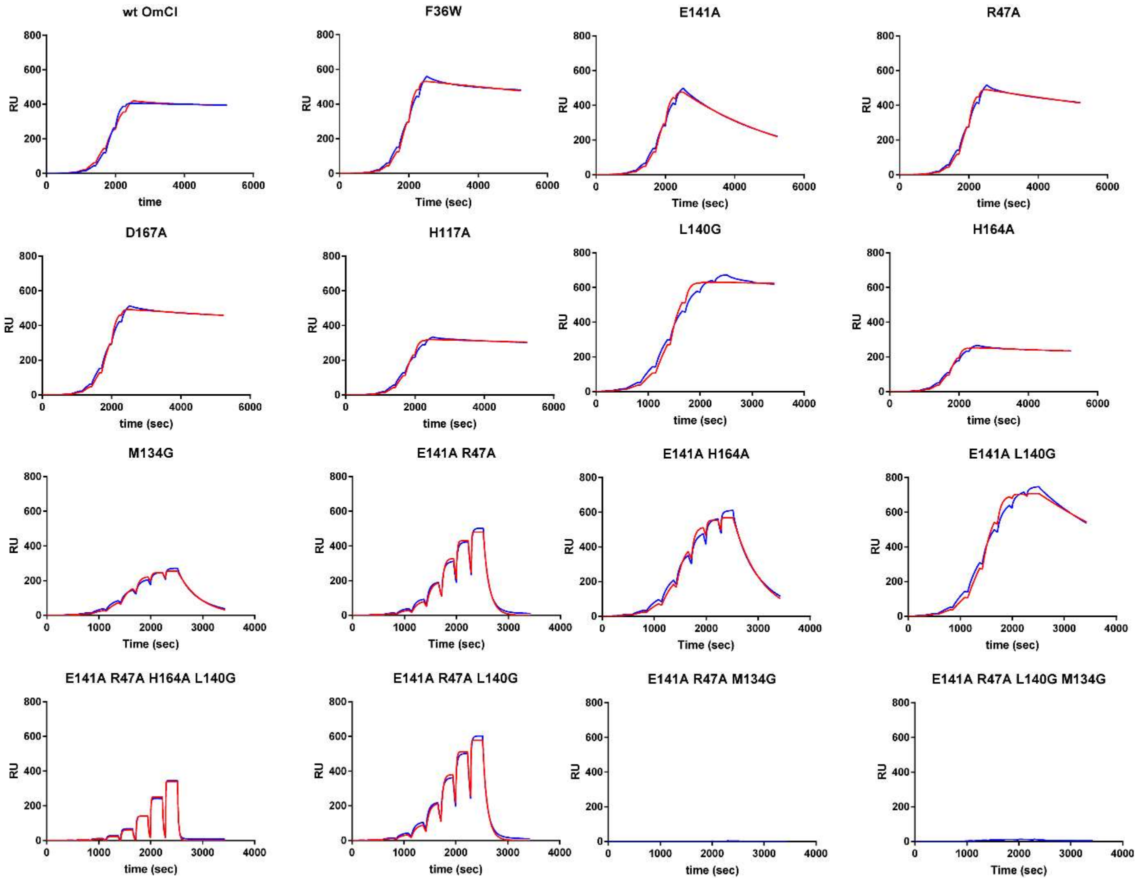

Figure S1. Example Sensorgrams from single cycle kinetics experiments, fitted with a one site binding model. The sensorgrams are shown in blue and the fit in red. The mutants show an accelerated k_{off} during the dissociation phase.

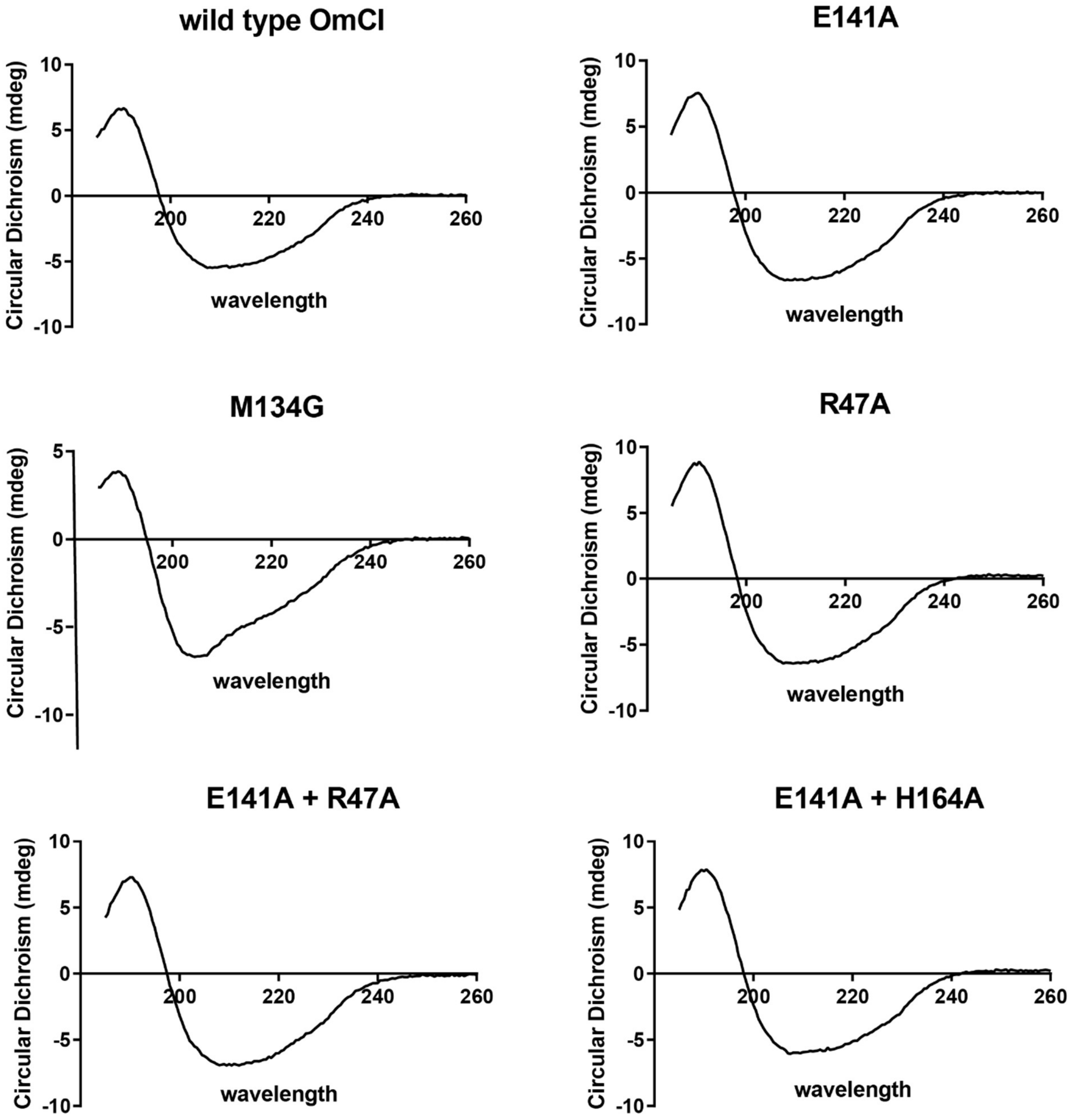

Figure S2. CD traces of OmCI proteins. All mutants show similar profiles by CD apart from M134G, which shows a marked loss of structure at 217nm consistent with a loss of β sheet. A reduction in the positive peak is also observed suggesting a loss of overall secondary structure.

Table S1
DSC data for OmCI proteins. Data from *n=1* experiment.

Mutation	Tm (ºC)	ΔTm (ºC)	ΔH (Cal/M)	ΔΔH (% refolded)
wt OmCI	64.39 ± 0.015	-	6.2E+04	-
wt OmCI refold	64.47 ± 0.016	+0.1	4.5E+04	71.7
R47A	59.56 ± 0.015	-4.8	4.9E+04	-
R47A refold	59.44 ± 0.012	-5.0	3.4E+04	69.1
E141A	61.08 ± 0.011	-3.3	5.7E+04	-
E141A refold	62.11 ± 0.011	-2.3	3.5E+04	61.7
H164A	62.8 ± 0.013	-1.6	9.2E+04	-
H164A refold	62.63 ± 0.011	-1.8	5.3E+04	57.7
E141A/ R47A	67.46 ± 0.011	+3.1	1.0E+05	-
E141A/ R47A refold	68.02 ± 0.006	+3.6	5.5E+04	54.1
E141A/ H164A	62.64 ± 0.015	-1.8	9.9E+04	-
E141A/ H164A refold	62.61 ± 0.001	-1.8	4.8E+04	47.8
M134G	56.18 ± 0.001	-8.2	4.6E+04	-
M134G refold	56.94 ± 0.001	-7.5	1.1E+04	23.9

Table S2
Multi cycle kinetics data summary table. Data from *n=4* experiments unless stated.

	k_{on} (1/Ms)	k_{on} 95% CI	k_{off} (1/s)	k_{off} 95% CI	K_D (M)
wt OmCI	5.88E+05	3.60E+05	<1.0E-05	-	<100pM
E141A	4.81E+05	2.28E+05	3.04E-04	2.20E-05	7.09E-10
E141A R47A	3.01E+06	2.96E+06	3.18E-02	2.57E-02	1.31E-08
E141A H164A*	1.13E+06	4.18E+05	4.06E-03	1.16E-03	4.09E-09

*average from *n=3* experiments

Multicycle kinetics data from individual occasions

n=1

	k_{on} (1/Ms)	K_{on} SE	k_{off} (1/s)	K_{off} SE	KD (M)
OmCI WT	3.84E+05	5.31E+01	<1.0E-05	-	<100pM
OmCI E141A	3.67E+05	9.25E+01	3.07E-04	2.07E-08	8.37E-10
OmCI E141A R47A	9.86E+05	8.49E+02	1.78E-02	5.49E-09	1.80E-08
OmCI E141A H164A	8.55E+05	4.18E+03	4.38E-03	6.37E-08	5.13E-09

n=2

	k_{on} (1/Ms)	K_{on} SE	k_{off} (1/s)	K_{off} SE	KD (M)
OmCI WT	4.00E+05	6.00E+01	<1.0E-05	-	<100pM
OmCI E141A	3.43E+05	3.08E+02	3.07E-04	2.30E-07	8.96E-10
OmCI E141A R47A	1.19E+06	1.33E+03	2.09E-02	2.25E-05	1.76E-08
OmCI E141A H164A	9.13E+05	4.38E+03	4.89E-03	2.41E-05	5.35E-09

n=3

	k_{on} (1/Ms)	K_{on} SE	k_{off} (1/s)	K_{off} SE	KD (M)
OmCI WT	1.14E+06	5.79E+02	<1.0E-05	-	<100pM
OmCI WT E141A	8.30E+05	3.00E+02	3.28E-04	1.68E-07	3.95E-10
OmCI E141A R47A	2.43E+06	2.50E+03	1.76E-02	1.70E-05	7.26E-09
OmCI E141A H164A	1.62E+06	1.66E+03	2.92E-03	2.45E-06	1.80E-09

n=4

	k_{on} (1/Ms)	K_{on} SE	k_{off} (1/s)	K_{off} SE	KD (M)
OmCI WT	4.32E+05	6.36E+01	<1.0E-05	-	<100pM
OmCI E141A	3.86E+05	8.19E+02	2.73E-04	4.04E-07	7.07E-10
OmCI E141A R47A	7.44E+06	3.19E+05	7.11E-02	3.07E-03	9.55E-09
OmCI E141A H164A*	ND	ND	ND	ND	ND

*Curve rejected.

Supplementary Table 3

Alternative pathway activation ELISA. Data from n=3 experiments.

Mutant	$\bar{x}$ pIC50	pIC50 range (n=3)	$\bar{x}$ Hill Slope	$\bar{x}$ Emax (%)
wt OmCI	≤ 9.0	9.0-9.1	>5	99.8
R47A	≤ 9.0	8.9-9.1	3.9	99.1
E141A	≤ 8.9	8.9	2.4	98.7
H164A	≤ 8.8	8.8	2.5	100.2
E141A/ R47A	8.0	8.0-8.1	0.9	94.7
E141A/ H164A	8.5	8.5-8.6	1.3	97.6

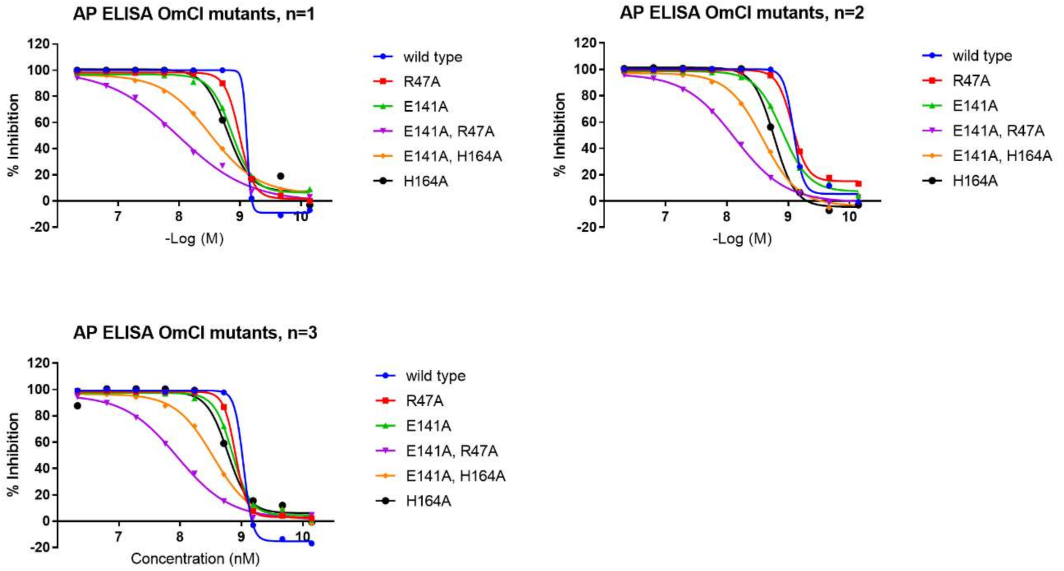

Supplementary Figure 3 Inhibiton of MAC formation in an Alternative Pathway ELISA. The ability of the OmCI mutants to inhibit formation of the MAC was tested by ELISA. The wt protein and single mutants are potent inhibitors and display steep hill slopes. All proteins show full efficacy with Emax values ~100%.

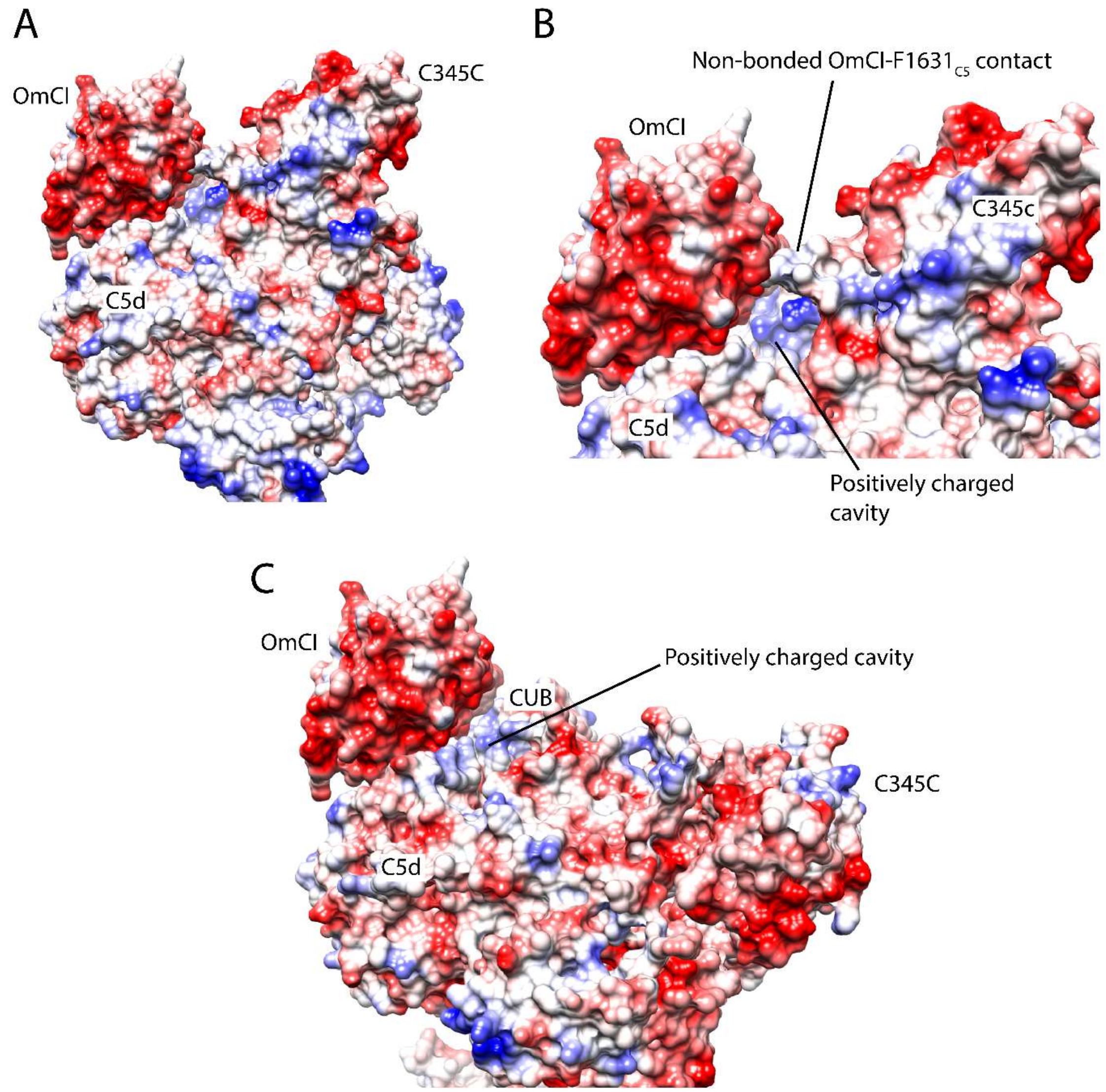

Figure S4. Surface charges of C5 and OmCI. Panel A shows the C5d-CUB-MG8 superdomain of C5 in complex with OmCI (PDB code: 5HCC), the surface is coloured by charge, with blue denoting areas of positive charge and negatively charged areas shown in red. OmCI is predominantly negatively charged. Panel B shows a close-up view of OmCI and C345c showing the non-bonded contact between the two, with a positively charged cavity visible between C5d and CUB domains. Panel C shows the C5d-CUB-MG8 superdomain from the apo C5 structure (PDB code 3CU7) in place of the superdomain from the OmCI-RaCI-C5 structure used in panels A and B. The positively charged cavity is accessible to OmCI when C345C is in the 'down position'.

Amino Acid Sequence for OmCI N-terminal AVI-10xHis

GGSHHHHHHHHHHGSGSENLYFQGSGSASSGLNDIFEAQKIEWHEDSESDCTGSEPVDAFQAFSEGKEA
YVLVRSTDPKARDCLKGEPAGEKQDNTLPVMMTFKNGTDWASTDWTFTLDGAKVTATLGNLTQNREVVY
DSQSHHCHVDKVEKEVPDYEMWMLDAGGLEVEVECCRQKLEELASGRNQMYPHLKDC

Figure S5. Amino acid sequence of OmCI constructs. The amino acid sequence of the final OmCI constructs is shown above. The residues comprising the N-terminal tags are coloured as follows: the 10x Histidine tag in green, a TEV cleavage site in pink and an AVI site in yellow. The residues mutated in the study are coloured cyan.
